# Scattering matrix imaging pulse design for real‐time respiration and cardiac motion monitoring

**DOI:** 10.1002/mrm.27884

**Published:** 2019-07-17

**Authors:** Sven H. F. Jaeschke, Matthew D. Robson, Aaron T. Hess

**Affiliations:** ^1^ Oxford Centre for Clinical Magnetic Resonance Research, Division of Cardiovascular Medicine, Radcliffe Department of Medicine University of Oxford Oxford United Kingdom; ^2^ Perspectum Diagnostics Oxford United Kingdom; ^3^ BHF Centre of Research Excellence University of Oxford Oxford United Kingdom

**Keywords:** cardiac self‐gating, cardiovascular magnetic resonance, dual gating, parallel transmit, respiratory motion, ultra‐high field MRI

## Abstract

**Purpose:**

The scattering matrix (S‐matrix) of a parallel transmit (pTx) coil is sensitive to physiological motion but requires additional monitoring RF pulses to be measured. In this work, we present and evaluate pTx RF pulse designs that simultaneously excite for imaging and measure the S‐matrix to generate real‐time motion signals without prolonging the image sequence.

**Theory and Methods:**

Three pTx waveforms for measuring the S‐matrix were identified and superimposed onto the imaging excitation RF pulses: (1) time division multiplexing, (2) frequency division multiplexing, and (3) code division multiplexing. These 3 methods were evaluated in healthy volunteers for scattering sensitivity and image artefacts. The S‐matrix and real‐time motion signals were calculated on the image calculation environment of the MR scanner. Prospective cardiac triggers were identified in early systole as a high rate of change of the cardiac motion signal. Monitoring accuracy was compared against electrocardiogram or the imaged diaphragm position.

**Results:**

All 3 monitoring approaches measure the S‐matrix during image excitation with quality correlated to input power. No image artefacts were observed for frequency multiplexing, and low energy artefacts were observed in the other methods. The accuracy of the achieved prospective cardiac gating was 15 ± 16 ms for breath hold and 24 ± 17 ms during free breathing. The diaphragm position prediction accuracy was 1.3 ± 0.9 mm. In all volunteers, good quality cine images were acquired for breath hold scans and dual gated CINEs were demonstrated.

**Conclusion:**

The S‐matrix can be measured during image excitation to generate real‐time cardiac and respiratory motion signals for prospective gating. No artefacts are introduced when frequency division multiplexing is used.

## 1 INTRODUCTION

For many cardiovascular MR applications, physiological motion and a reliable monitoring of such remains the major challenge for image quality. Long, free‐breathing image acquisitions suffer from blurring and ghosting artefacts that are induced by respiratory motion.^1^ As simple breath holds limit the acquisition time (<20 s) and are often not feasible for patients, diaphragm navigators are a common solution for respiratory gated and tracked acquisitions.^2^ Those navigators require additional, low‐resolution images that will subsequently prolong the overall scan time. External devices, such as respiratory bellows, can measure chest movements but will increase patient setup time and need careful adjustments for a reliable motion tracking.^3^, ^4^

The electrocardiogram (ECG) has been used extensively to monitor the heart activity, however, this can be cumbersome in some patients to setup because of the magneto‐hydrodynamic effects that overlay the electric fields from the heart and disrupt the ECG signal.^5^, ^6^ This is particularly apparent at high field. Other external devices that are independent to the electrical activity of the heart are based on finger plethysmography, Doppler ultrasound,^7^ or on heart tones (phonocardiograms).^8^

Alternative methods estimate cardiac and respiratory motion from surrogate signals that are derived from the continuous acquisition of the k‐space centre,^9^, ^10^, ^11^, ^12^, ^13^, ^14^, ^15^, ^16^, ^17^ from a region‐of‐interest in the image domain,^18^, ^19^ or from navigator echoes^20^, ^21^, ^22^, ^23^ that are added to the imaging sequence. Some of these methods have sequence design constraints; others are limited to a specific k‐space sampling trajectory.

A different approach assesses motion from changes in the load of the RF coils that are weighted by the coil profile and the conductivity of the tissue. This effect has been observed in receiver arrays by monitoring the noise characteristics^24^, ^25^ or using a reference transmitter (called a “pilot tone navigator”).^26^, ^27^, ^28^, ^29^ For a transmit RF‐coil, this effect was used to show respiratory and cardiac influence early on by Buikman et al.^30^ For transmit arrays or parallel transmit (pTx), pickup coils that measure the currents in the coil elements can be used to monitor respiratory motion.^31^, ^32^

The scattering matrix (S‐matrix) of a pTx coil has been shown to be effective at monitoring cardiac motion,^33^ but measuring the S‐matrix requires additional monitoring RF pulses. However, a subset of scattering information can be measured from excitation RF pulses alone and is termed the scattering coefficients. The scattering coefficient is a vector with a length of the number of transmit channels, whereas the S‐matrix is a matrix of the number of transmit channels squared. The scattering coefficient is simple to measure but suffers from being transmit state‐dependent. Previous work has shown that the motion signals derived from the S‐matrix are superior to the scattering coefficient for cardiac gating,^34^ resulting in higher SNR and lower cardiac trigger variation.

In this work, methods to monitor the S‐matrix simultaneously with the image excitation are developed and assessed to maintain the high SNR derived from S‐matrix measurements and bridge the gap between previously separated monitoring and imaging RF pulses. The goal is an RF pulse that excites an image slice and monitors the transmit S‐matrix to generate a real‐time motion signal during imaging. This is particularly useful as it will not prolong the image sequence, could be used with any k‐space sampling trajectory and is synchronized with the data acquisition. Three different RF pulse designs are evaluated. The quality of respiratory and cardiac motion signals, derived from these S‐matrix measurements, are compared to the use of scattering coefficients. Finally, a prospective dual gating system is evaluated in healthy volunteers at 7T.

## 2 THEORY

### 2.1 Scattering matrix measurements

The scattering of a pTx coil can be used to estimate respiratory and cardiac motion when dedicated pulse sequences are implemented. The basis of these approaches are described in more detail in Jaeschke et al^35^ and Hess et al.^36^ In brief, independent RF monitoring pulses enable the identification of the fractional returned voltages $V_{i,j,\text{ret}}$ on each channel *i*, which originate from the forward voltage ($V_{j,\text{fwd}}$) on channel *j*, to calculate the S‐matrix of the pTx coil.

(1) $$V_{i,j,\text{ret}\;}=S_{i,j}V_{j,\text{fwd}\;}.$$

We assumed that cardiac and respiratory changes are linear and independent, and therefore we model the time‐resolved S‐matrix S(t) with 3 additive terms: $S_{0}$, which is static S‐matrix and represents the coil properties and the non‐movable tissue, $\Delta S_{\text{resp}}\left(t\right)$, which represents respiratory induced changes, and $\Delta S_{\text{cardiac}}\left(t\right)$, which reflects the change resulting from cardiac motion.

### 2.2 Pulse design for S‐matrix measurements

To measure the S‐matrix during image acquisition an S‐matrix measurement RF pulse is combined with a slice selective excitation RF pulse. Three methods for simultaneous S‐matrix measurement were identified from RF‐communications engineering^37^ and include (1) frequency division multiplexing (FDM), (2) time division multiplexing (TDM), and (3) code division multiplexing (CDM). Here, we denoted *f*(*t*) as the image excitation RF pulse function (e.g., truncated sinc), *g*(*t*) as the monitoring RF pulse function, and *F*(*f*) and *G*(*f*) as their respective Fourier transforms

(2) $$a\;f\left(t\right)+b\;g\left(t\right)\;of\;a\;F\left(\omega \right)+b\;G\left(\omega \right),$$

with different scaling factors *a* and *b*.

Several conditions apply for the S‐matrix monitoring schemes. The RF pulses should be orthogonal to each other on each channel, should not introduce image artefacts, should not increase the noise in the image, and should not increase the duration of the excitation pulse or alter the sequence timing. A minimal SAR burden is preferable. The following implementations are considered for these conditions.

#### 2.2.1 Frequency division multiplexing

Frequency division multiplexing (FDM) allocates different frequency bands for each transmit channel k, where *N* is the total number of channels. A frequency spacing Δf separates each channel. For harmonic sinusoids, the minimum Δf is defined by the pulse length (T) as 1/T, in this work 10/T was used (10 kHz for a 1 ms RF pulse).

(3) $$s\left(t\right)=f\left(t\right)+b\sum_{k=0}^{N-1}g\left(t\right)e^{-j2\pi \left(f_{\mathrm{slc}}+f_{\mathrm{off}}+k\Delta f\right)}\;of\;S\left(f\right)=F\left(f\right)+b\sum_{k=0}^{N-1}G\left(f_{\mathrm{slc}}+f_{\mathrm{off}}+k\Delta f\right).$$

A monitoring frequency offset $f_{\mathrm{off}}$ relative to the slice centre carrier frequency $f_{\mathrm{slc}}$ of the imaging RF pulse is used to avoid interference with the main imaging RF pulse and to avoid excitation of the object. For a given slice‐select gradient, $f_{\mathrm{off}}$ is chosen so that any off‐resonance excitation would be outside of the FOV of the coil elements. The total bandwidth *B* of the monitoring RF pulse is limited by the hardware specifications of the MRI system.

In this work, fermi‐shaped monitoring pulses *g*(*t*) with the same length as the excitation RF pulse were used with a monitoring offset frequency of 100 kHz and frequency spacing of 10 kHz for each transmit channel.

#### 2.2.2 Time division multiplexing

(4) $$s\left(t\right)=af\left(t\right)+b\sum_{k=0}^{N-1}g\left(t+kT_{g}\right)\;of\;S\left(f\right)=aF\left(f\right)+b\;G\left(f\right)\sum_{k=0}^{N-1}e^{j2\pi fk\left(\frac{1}{T_{g}}\right)}.$$

**Figure 1:**
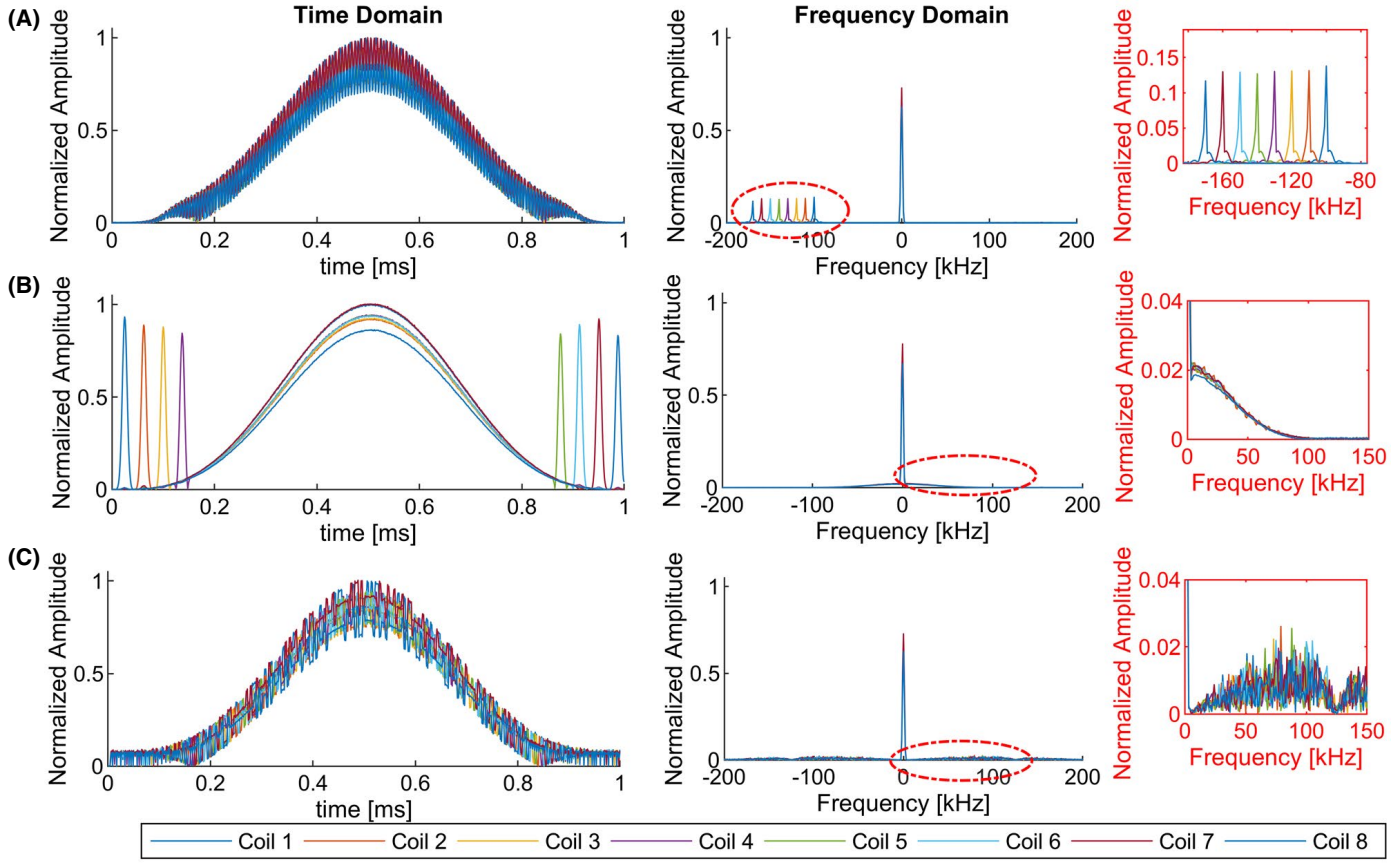
Example of overlaid imaging and S‐matrix RF pulses using (A) FDM, (B) TDM, and (C) CDM. The normalized magnitude of the complex RF waveform, as measured by the DICOs of the MR scanner, are shown in the left column with the respective Fourier‐transforms in the right columns

Time division multiplexing (TDM) allocates different timeslots for each channel and transmits a monitoring pulse on 1 channel at a time. In this work, short Gaussian‐shaped monitoring pulses (T_g_ = 35 μs) were applied at the beginning and end of the truncated sinc‐shaped excitation RF pulse *f*(*t*). Therefore, they do not limit the peak RF amplitude and decrease interference with imaging RF pulse. The RF‐energy is spread in the frequency domain (Figure 1C).

#### 2.2.3 Code division multiplexing

(5) $$s\left(t\right)=af\left(t\right)+b\sum_{k=0}^{N-1}g\left(t\right)\;\cdot \;c_{k}\left(t\right)\;of\;S\left(f\right)=aF\left(f\right)+b\sum_{k=0}^{N-1}G\left(f\right)*C_{k}\left(f\right)\text{where}\;\;c_{k}\;⊥\;c_{k+1}.$$

Where TDM and FDM enable orthogonality to the imaging RF pulse and encoding by a clear separation in time and frequency domain respectively, code division multiplexing (CDM) uses the full RF pulse length and the full transmit and/or receive bandwidth for all channels. Unique encoding patterns *c_k_* enable channel identification.

In this work, we have used pseudo‐random noise (PRN) encoding. Ultra‐short (td = 4 μs), complex, rectangular RF‐sub pulses were alternated in a pseudo‐random fashion in the real and imaginary domain ([Real, Imaginary] = [1,0],[0,1],[0,−1],[−1,0]) to excite a broad bandwidth and to create pseudo‐random noise in the frequency domain (Figure 1B). Each transmit channel k was given a distinct, pseudo‐random code pattern c_k_.

To avoid image artefacts, each RF sub pulse was followed by its negative to suppress the energy at the central frequency. The randomness of the encoding pattern was restricted so that all 4 states occur within 8 consecutive RF sub pulses. Remaining out‐of‐slice magnetization is expected to appear as noise (Figure 1C).

## 3 METHODS

### 3.1 Cardiac and respiratory motion estimation

A sagittal image was continually acquired using the chosen S‐matrix monitoring scheme during a training period of 72 s (0.9 × 5 × 5 mm^3^; TR/TE = 4/1.51 ms; temporal resolution, 240 ms). Calibration was carried out offline in MATLAB (The MathWorks, Natick, MA). An independent component analysis (ICA) was used to extract the cardiac signal from the S‐matrix and the scattering coefficient measurements, as in Jaeschke et al.^33^ The polarity of the cardiac signal was determined on the dominant peak in the signal to be positive.

For respiratory motion calibration from the images, a canny edge detection algorithm, using a Sobel operator, defined the position of the diaphragm. The measured diaphragm positions were up‐sampled in the time domain to the RF pulse sampling rate using a smoothing b‐spline interpolation. A linear regression model was trained on the diaphragm position using the S‐matrix as well as the scattering coefficients, similar to previously described in Hess et al.^36^ A ℓ2‐norm and ℓ1‐norm penalty of the regression coefficients was added to the regression model to reduce the variance of the linear regression coefficients and to increase the robustness of the diaphragm position estimation.

A second calibration data set was acquired to calculate the RMS error between the measured diaphragm position and the offset‐corrected, predicted diaphragm position using both the S‐matrix and scattering coefficients measurements.

Both the coefficients of the linear regression model and the estimated cardiac de‐mixing vector were saved to a file to be used to calculate real‐time motion signals on the image calculation environment (ICE) of the MR scanner for prospective gating.

Prospective cardiac gating was implemented using a Kalman filter,^38^ with a constant velocity model to estimate the magnitude of the cardiac vector and its rate of change (with a covariance of the observation of R = 0.2 and a covariance of the process of Q = 0.001). A cardiac trigger is defined as the rate of change of the cardiac signal increasing above 4 (found empirically). A refractory period (close to that of the human heart) of 250 ms was used to avoid false–positive trigger and to increase the robustness of the peak detection algorithm.

### 3.2 In vivo experiments and data acquisition

Measurements were made on a 7T MRI Scanner (Magnetom 7T, VB17, Step 2.3, Siemens, Erlangen, Germany) with an 8‐channel, dipole cardiac parallel transmit/receive coil (MR Coils Zaltbommel, Netherlands). Seven healthy volunteers were recruited according to our institution's ethical practices (3 female, 4 male, age range 24–38 y, body mass index = 21.5 ± 1.2) for prospective cardiac‐gated MRI. Additional respiratory motion and monitoring power evaluation was carried out for 5 of these participants.

The forwarded and returned waveforms were measured using the systems directional couplers (DICOs) that are built into the transmission line of each channel and that split off a small portion of the signal. The DICOs are part of the internal safety monitoring of the scanner and can be used to measure the specific absorption rate (SAR).

Different power levels of S‐matrix monitoring pulses were assessed for imaging with a breath hold cine image acquisition using a 2D gradient recalled echo (GRE) sequence (1.4 × 1.4 × 5 mm^3^; TR/TE = 4.57/1.51 ms; a generalized auto‐calibrating partial parallel acquisition factor of 2). The S‐matrix was measured with 3 different monitoring schemes using modified RF pulses as described above. Artefact images were created with the main imaging RF pulse switched off. For the power level evaluation, these images were retrospectively cardiac‐gated using the extracted cardiac signal form the S‐matrix measurements as in Jaeschke et al.^35^ Power levels were calculated on the measured forwarded waveforms as pulse energy per TR assuming a 50Ω regime for each channel.

The quality of the extracted cardiac signal was estimated using the SNR, defined as the mean peak amplitude of the cardiac signal divided by the noise. The noise in the cardiac signal was calculated as the SD of the difference of a filtered (Savitzky‐Golay filter)^39^ cardiac signal from the unfiltered cardiac signal. For comparison purposes, an additional cardiac signal was calculated using the scattering coefficient method^40^ that is based on the reflections from the imaging pulse alone, and its SNR was determined.

The effect on SAR of the added RF power of the 3 multiplexing methods was evaluated in terms of 2 different SAR limiting modes. The first mode is a worst‐case power limit, defined as the maximum 6‐min average power that is allowed on each channel, such that for any parallel transmit configuration the first level controlled limit of 20 W/kg will not be exceeded. This mode approves the use of our coil with a power limit of 3.8 W on each channel. The second mode is less conservative and calculates the local SAR produced by a specific pTx waveform. To evaluate the added local SAR of the monitoring pulses, E‐fields for each transmitter were calculated using Sim4Life (ZMT Zurich MedTech) on the virtual human Duke (IT'IS Foundation). Dukes local SAR was simulated for the cine sequence described in 50 different RF shim configurations, and for each multiplexing method, both with and without added S‐matrix monitoring with maximum monitoring power.

2D GRE CINE images were acquired during breath hold and free‐breathing using the new prospective, dual‐gating method based on simultaneous S‐matrix measurements with FDM S‐matrix monitoring with an amplitude of 15–20% of that of the image excitation RF pulse. A linear k‐space encoding was used and respiratory motion states were evaluated once after each cardiac trigger. Free‐breathing images were acquired during end‐expiration with an acceptance window of 5 mm.

For all measurements, ECG triggers were recorded for comparison of the accuracy of the proposed methods. Trigger events were assumed to occur only 1 time during a normal heart cycle. Missed (false negatives) or additional triggers (false positives) were manually assessed by overlaying the ECG and the extracted cardiac signal.

A generalized linear mixed model was used to test the influence of the 3 different monitoring methods and of the power level on the cardiac SNR. A multiple linear regression analysis evaluated the impact of the body–mass index of the participants.

## 4 RESULTS

### 4.1 Scattering matrix measurements: Monitoring power and image artefacts

**Figure 2:**
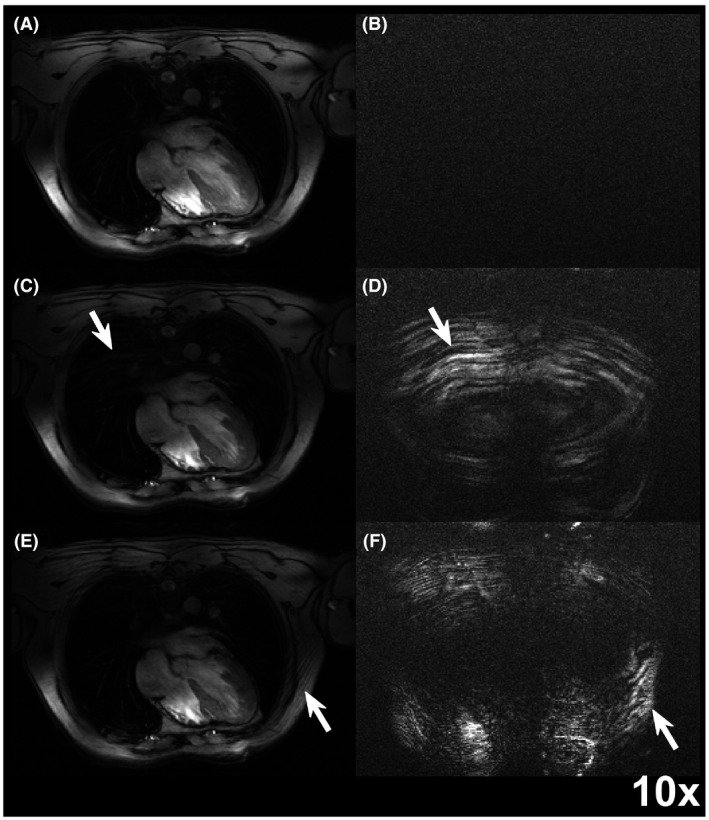
S‐Matrix gated images using (A) FDM, (C) CDM, and (E) TDM during breath hold using the highest monitoring power level. In (B), (D), and (F), images with only monitoring RF pulses and the imaging RF pulse switched off are shown. Artefact introduction for CDM and TDM monitoring schemes can be observed in (C)‐(D) and (E)‐(F), respectively

The S‐matrix was calculated for all tested monitoring RF pulses. All 3 different monitoring schemes enabled retrospective cardiac‐gated image reconstruction using the cardiac signal derived from the calculated S‐Matrices. CDM and TDM monitoring show some artefacts around the anterior and posterior chest walls. FDM had no visible artefacts. Typical example images are shown in Figure 2 for 1 subject.

**Figure 3:**
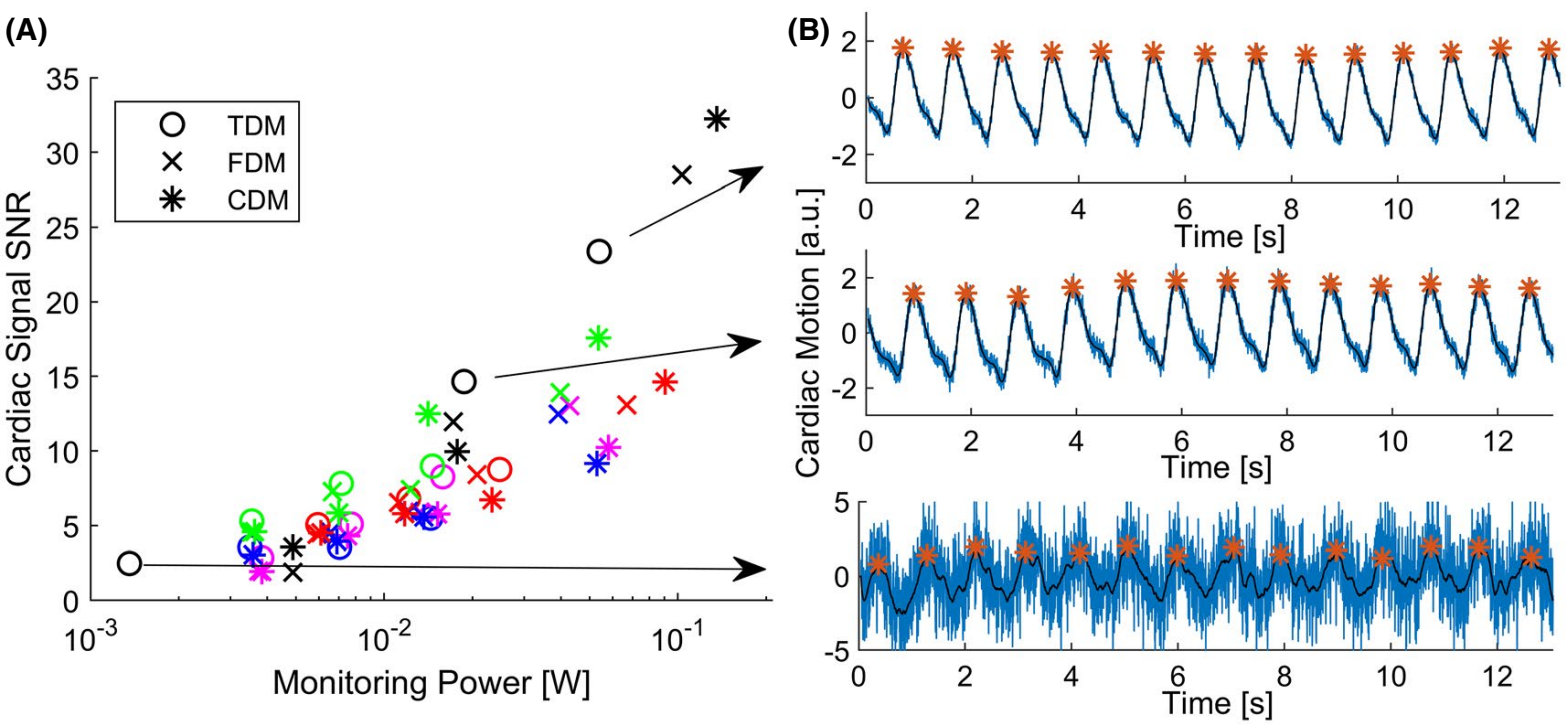
SNR for different monitoring energy levels using S‐matrix monitoring with FDM, TDM, and CDM in 5 healthy subjects show with colored markers in (A). An example of the cardiac signals derived from different S‐matrix monitoring power level are shown in (B) using TDM

The estimated cardiac signals had an SNR of 1.1–31.8 for the different power levels that had RF amplitudes from 1.7% up to 20% of that of the imaging RF pulse (Figure 3). Using only the scattering coefficient of the normal imaging RF pulse without an additional monitoring, an average cardiac SNR of 3.6 ± 1.2 can be achieved. No statistically significant differences related to the different S‐matrix monitoring methods and cardiac SNR were observed (*F*(3,44) = 2.7, *P* = 0.08). Inter‐subject variance in cardiac SNR was unrelated to the assessed, small range of body mass indices in this study (*P* = 0.833).

The maximum monitoring power level for FDM, CDM, and TDM, added 0.09 W, 0.12 W, and 0.05 W on each channel respectively. Compared to our conservative 6‐min average power limit (3.8 W), the monitoring power only contributed 2.5%, 3.3% and 1.4% for FDM, CDM and TDM respectively. The local SAR simulations in Duke ranged from 0.08–0.13 W/kg for FDM, 0.11–0.17 W/kg for CDM, and 0.06–0.08 W/kg for TDM for the different shim configurations.

### 4.2 Physiological motion estimation and prospective image acquisition

The diaphragm position was estimated with a RMS error of 1.3 ± 0.9 mm using the S‐matrix measurements (FDM monitoring) and 2.3 ± 2.3 mm using scattering coefficients. The accuracy of diaphragm position estimation is reduced for larger diaphragm amplitudes and when the time between training and prediction data sets increases.

The large majority (>99%) of cardiac cycles were correctly identified using the S‐matrix FDM method in an online prospective data analysis. Two free‐breathing acquisitions had false positive trigger (7 out of 148 and 3 out of 196 heart beats). The ECG had 2 missed triggers in 1 data set. These false positives were excluded for further data analysis. The prospective cardiac trigger had a mean temporal delay of 158 ± 63 ms compared to ECG R‐wave detection during breath hold and a mean temporal delay of 146 ± 56 ms during free breathing. The SD with respect to ECG in cardiac trigger time was 15 ± 16 ms for breath hold and 24 ± 17 ms during free breathing.

**Figure 4:**
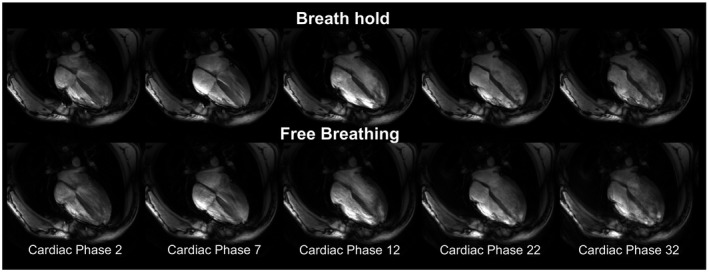
Four‐chamber view of a prospectively, cardiac‐gated 2D GRE CINE using the proposed method during breath hold and free breathing. Five cardiac phases are shown, each capturing a time window of 33 ms of the cardiac cycle. From left to right, the images show early ventricular systole to end‐diastole of the cardiac cycle

Free‐breathing CINEs, gated to end‐expiration using the dual respiratory and cardiac prospective S‐matrix monitoring, were reconstructed online and demonstrated no observable respiratory artefacts in 3 of 5 CINEs. Some blurring and residual motion artefacts around the chest‐wall at end‐diastole appeared for the other data sets. An example of prospective gated Cine during breath hold and free breathing is shown in Figure 4 with all other subjects shown in Supporting Information Videos [Link], [Link], [Link], [Link], [Link], [Link], [Link], [Link], [Link], [Link], [Link], [Link].

## 5 DISCUSSION

This work demonstrated that 3 methods can be used to measure the S‐matrix of a pTx coil at the same time as image excitation and as such does not increase the sequence duration. CDM and TDM introduced small image artefacts, and FDM presented no measurable artefacts. The cardiac signals can be estimated for all tested monitoring amplitudes and methods with the SNR of the cardiac signal being correlated to the power of the monitoring pulse. This indicates that the bandwidth and/or frequency or amplitude of the monitoring scheme have no distinguishable effect on the quality of the measured signals.

For CDM, the RF‐sub pulses should be as short as possible to spread out the energy in the frequency spectrum and away from the FOV of the object. We have found that a minimum of 4 μs enabled the RF amplifier to reach the requested RF amplitudes, giving a white noise bandwidth of 250 kHz. We expect the artefacts from the CDM scheme would be reduced for shorter RF sub pulses. The use of a wider monitoring bandwidth does not seem to have improved the S‐matrix calculation. In this work, we did not evaluate changing the code for every RF pulse, it is conceivable that such a scheme would produce incoherent signal from shot to shot and mitigate the observed artefacts.

The TDM scheme has been applied during the imaging RF pulse, but each monitoring pulse could be applied at any time during the image sequence. Although TDM has shown good results for varying power levels, unwanted excitation has introduced small artefacts. Alternatively, TDM could be applied during crusher gradients to avoid image interference but would reduce its general applicability.

FDM does not introduce measurable artefacts by setting the monitoring frequency outside the range of the imaging object for a given slice‐select gradient. Compared to CDM and TDM, the monitoring bandwidth is clearly separated from the imaging RF pulse bandwidth, and any possible spin excitation appears outside the field of view of the coils. However, the overall monitoring bandwidth needs to be within the range of the MR system specifications and therefore limits the maximum monitoring frequency separation and offset.

The RF power for additional S‐matrix monitoring is very small compared to our systems worst‐case SAR power limit of 3.8 W per channel. The impact on local SAR is again minimal, with the highest increase found in CDM monitoring being 0.9% of the 6 min SAR limit in first level controlled mode of 20 W/kg.

Free‐breathing image acquisitions were possible and gating is comparable to previous, retrospective work. The current prospective data acquisition used a linear k‐space encoding where the respiratory state was updated every heart cycle. This may have caused some of the artefacts when the respiratory state changed early with the heart cycle (compare Supporting Information Videos S5,S10). This technology can also be used to monitor the compliance of patients to breath holds when no respiratory gating is needed.

The motion signals might be sensitive to the number of evaluated pTx channels and coil elements. Future work will test the applicability in lower field strengths (i.e., at 3T MRI with commercially available 2‐channel pTx systems).

The presented monitoring approach is MR‐sequence independent as it can be overlaid with any RF excitation pulse and is therefore suitable for all k‐space trajectories. In comparison to navigator echoes and image‐based motion estimation, it does not alter the sequence timing or prolongs the image acquisition. Further, it has the potential to reduce the patient preparation time as no additional monitoring hardware, such as ECG or respiratory bellows, is needed. In contrast to pilot‐tones monitoring, this method requires RF pulse modification but no additional hardware.

## 6 CONCLUSION

In conclusion, a high SNR in the extracted motion signals can be observed that enabled prospective cardiac gating, simultaneous respiratory motion monitoring, and high quality, online image reconstruction with only a low, extra RF‐power burden.

## Supporting information


**VIDEO S1** Four‐chamber view of a prospectively, cardiac‐gated 2D GRE CINE using the proposed method during breath hold on volunteer 1

**VIDEO S2** Four‐chamber view of a prospectively, dual‐gated 2D GRE CINE using the proposed method during free breathing on volunteer 1

**VIDEO S3** Four‐chamber view of a prospectively, cardiac‐gated 2D GRE CINE using the proposed method during breath hold on volunteer 2

**VIDEO S4** Four‐chamber view of a prospectively, cardiac‐gated 2D GRE CINE using the proposed method during breath hold on volunteer 3

**VIDEO S5** Four‐chamber view of a prospectively, dual‐gated 2D GRE CINE using the proposed method during free breathing on volunteer 3

**VIDEO S6** Four‐chamber view of a prospectively, cardiac‐gated 2D GRE CINE using the proposed method during breath hold on volunteer 4

**VIDEO S7** Four‐chamber view of a prospectively, dual‐gated 2D GRE CINE using the proposed method during free breathing on volunteer 4

**VIDEO S8** Four‐chamber view of a prospectively, cardiac‐gated 2D GRE CINE using the proposed method during breath hold on volunteer 5

**VIDEO S9** Four‐chamber view of a prospectively, cardiac‐gated 2D GRE CINE using the proposed method during breath hold on volunteer 6

**VIDEO S10** Four‐chamber view of a prospectively, dual‐gated 2D GRE CINE using the proposed method during free breathing on volunteer 6

**VIDEO S11** Sagittal view of a prospectively, cardiac‐gated 2D GRE CINE using the proposed method during breath hold on volunteer 7

**VIDEO S12** Sagittal view of a prospectively, dual‐gated 2D GRE CINE using the proposed method during free breathing on volunteer 7
